# Fruit Quality Assessments of Six Sweet Cherry Cultivars Grown in the Arid Northwest China

**DOI:** 10.3390/foods15162809

**Published:** 2026-08-12

**Authors:** Haochen Wang, Qin Wang, Yao Lu, Yameng Zheng, Siqing Dang, Yulin Fang, Jiangfei Meng

**Affiliations:** 1 Shaanxi Key Laboratory for Viti-Viniculture, College of Enology, Northwest A&F University, Xianyang 712100, China; whch2020@163.com (H.W.); lyluyao@nwafu.edu.cn (Y.L.); 09091340@nwafu.edu.cn (Y.Z.); dang@nwafu.edu.cn (S.D.); fangyulin@nwafu.edu.cn (Y.F.); 2 Key Laboratory of Fruit Tree Species Breeding and Cultivation in Xinjiang, Key Laboratory of Desert-Oasis Ecosystem Protection and Restoration of National Forestry and Grassland Administration, Institute of Economic Forestry, Xinjiang Uygur Autonomous Region Academy of Forestry Sciences, Urumqi 830000, China; 3 Akesu Observation and Research Station of Chinese Forest Ecosystem, Aksu 843000, China

**Keywords:** sweet cherry, odor activity value (OAV), varietal differences, quality evaluation, volatile compounds, esters, acids, terpenes, aldehydes

## Abstract

Sweet cherries are high-value commercial fruits whose aroma is a major determinant of fruit quality. This study investigated fruit quality, sugar and organic acid profiles, phenolic composition, and volatile compounds in six sweet cherry cultivars, namely ‘Hongdeng’, ‘Lapins’, ‘Summit’, ‘Rainier’, ‘Van’, and ‘Tieton’, cultivated in arid Kashgar, Xinjiang. Glucose and fructose were the predominant soluble sugars, whereas malic acid and citric acid were the principal organic acids. ‘Rainier’ and ‘Lapins’ exhibited contrasting organic acid accumulation patterns. A total of 56 volatile compounds were identified, with alcohols predominating, followed by esters, aldehydes, and acids. Significant cultivar-dependent differences were observed in total volatile content, soluble solids, fruit color, and firmness. ‘Rainier’ showed the highest total volatile concentration, highest soluble solids content, and highest L* and b* values, and was characterized by isobutanol, isoamyl alcohol, and benzaldehyde. ‘Tieton’ showed the greatest firmness and ‘Summit’ had highest a* value. ‘Tieton’ produced the largest fruits, ‘Hongdeng’ had softer flesh, and ‘Van’ contained high levels of octanoic acid. Odor activity value analysis identified four key aroma compounds. These findings support objective quality evaluation, cultivar selection, and quality management of sweet cherries in arid regions.

## 1. Introduction

Sweet cherry (*Prunus avium* L.) is an economically valuable and widely appreciated fruit worldwide. Consumer preference for this fruit is based on its attractive appearance, crisp texture, balanced sweet-sour taste, and rich nutritional profile, including vitamins, polyphenols, and potent antioxidant compounds [1]. Global sweet cherry production has increased consistently in recent decades, with China emerging as a major producer and consumer of sweet cherries. Various sweet cherry cultivars with distinct phenotypic and flavor traits have been bred and promoted, creating diverse market choices, while also increasing the requirements for objective quality evaluation and varietal improvement [2].

Fruit quality is a comprehensive concept determined by numerous interrelated indicators [3]. Key quality indicators of cherry fruit include fruit size, soluble solids content, titratable acidity, and skin color parameters. These attributes directly influence consumer acceptance and commercial value [2,3]. Among them, skin color is the most visually apparent trait and is closely associated with the accumulation of anthocyanins and other pigments. Consequently, it is widely used as an important indicator of fruit maturity, storage potential, and commercial grade [4]. In addition to its physicochemical properties, aroma is a core factor affecting the sensory quality and market competitiveness of cherries [5]. The unique and pleasant aroma of sweet cherries can be attributed to a complex mixture of volatile compounds, primarily esters, alcohols, aldehydes, terpenes, and ketones. These compounds provide sweet cherry fruit with characteristic fruity, floral, green, and sweet notes as well as form variety-specific aromatic fingerprints [5]. Notably, the climate conditions of the cultivation region affect the aroma and physicochemical properties of sweet cherries, and consequently their quality.

Many studies have analyzed the volatile components and quality traits of certain sweet cherry cultivars [6]. Villavicencio et al. (2021) indicated that climatic factors, including light, temperature, precipitation, and altitude, are important in the accumulation of aroma compounds in sweet cherry fruit [7], while Vavoura et al. (2015) revealed that the interaction between cultivars and climate results in pronounced differentiation in the aromatic fingerprint profiles of sweet cherries [8]. However, systematic and comprehensive evaluations that integrate physicochemical properties, volatile components, and odor activity values remain lacking, particularly for the main cultivated sweet cherry cultivars. Genetic differences result in considerable variations in appearance, texture, nutritional components, and aroma patterns among the cultivars. However, the key indicators and marker compounds that distinguish different sweet cherry cultivars remain to be identified.

Therefore, six sweet cherry cultivars widely cultivated in Kashgar, Xinjiang, namely Hongdeng, Lapins, Summit, Rainier, Van, and Tieton, were selected for analysis. This study aimed to systematically characterize the physicochemical quality attributes of six major sweet cherry cultivars cultivated in the arid region of Kashgar, including fruit size, firmness, soluble solids content, organic acid content, total phenolic content, soluble sugar content, and skin color parameters; comprehensively profile their volatile aroma compounds and identify the key odor-active compounds using odor activity value analysis; and elucidate the relationships between physicochemical properties and volatile profiles through multivariate statistical analysis to identify cultivar-specific quality markers. The study further sought to clarify the quality variation and aroma characteristics among the cultivars, establish a comprehensive quality evaluation system, and provide a scientific basis and supporting data for sweet cherry quality assessment, grading, breeding selection, and industrial development.

## 2. Materials and Methods

### 2.1. Chemicals and Reagents

All standards for volatile compounds with a purity of ≥97% were purchased from Sigma-Aldrich (Shanghai, China). Other chemical reagents, including D-gluconolactone and polyvinylpolypyrrolidone (PVPP), were sourced from Tianjin Kemel Chemical Reagent Co., Ltd. (Tianjin, China) and met or exceeded analytical grade standards.

### 2.2. Plant Materials and Growing Conditions

The study was conducted in Mixia Town, Shache County, Kashgar, Xinjiang, China, a typical arid region in northwestern China. The area is characterized by flat terrain, an average elevation of approximately 1200 m, a warm temperate continental arid climate, and sandy loam soil. The region has an annual mean temperature of 11.5–13.5 °C, annual precipitation of only 50–80 mm, annual sunshine durat ion of 2800–3000 h, and an effective accumulated temperature above 10 °C of 4200–4600 °C. During the 2025 growing season, the mean temperature increased from 9.8 °C in March to 27.2 °C in June. The mean maximum temperature was 35.0 °C in June, whereas the mean minimum temperature was 3.0 °C in March. Total precipitation during the growing season was 56.3 mm, with rainfall occurring mainly in April to June (Table S1 and Figure S1).

At the time of sampling, all trees were twelve years old. The trees were grafted onto vigorous rootstocks and trained primarily to an open-center form. The planting spacing was 3.0 m × 7.0 m. Supplemental irrigation was applied using a furrow irrigation system, with a total of five applications during the critical growth periods throughout the year, and an annual total irrigation volume of approximately 6000–7500 m^3^ ha^−1^. Well-rotted manure was applied annually in autumn at a rate of 30 t ha^−1^. No rain shelters or protective netting were installed in the orchard.

All six sweet cherry cultivars began budburst in late March. ‘Hongdeng’, ‘Van’, ‘Summit’ and ‘Tieton’ reached full bloom in early April, whereas ‘Lapins’ and ‘Rainier’ reached full bloom in mid-April. The selected fruits were uniform size, and the characteristic skin color of each cultivar. The skin was dark red in ‘Hongdeng’, ‘Lapins’, ‘Summit’, ‘Van’, and ‘Tieton’, and yellowish red in ‘Rainier’. In 2025, the fruit were harvested in batches from late May to early June. Immediately after harvest, the fruit samples were transported to the laboratory under refrigerated conditions and stored at −20 °C until subsequent quality analyses.

### 2.3. Determination of Basic Physicochemical Properties

Soluble solids content (°Brix): Ten sweet cherry samples were randomly selected. Tissue samples were collected from symmetrical equatorial positions of the fruits, mixed, and ground into a homogenate, which was then filtered through gauze to obtain clear fruit juice. The soluble solids content (°Brix) of sweet cherry juice was determined using a PAL-1 handheld refractometer (ATAGO, Tokyo, Japan). Prior to the measurement, the refractometer was calibrated with distilled water. Each sample was assayed in triplicate, and the final result was expressed as the mean value [9].

Conditions for the determination of firmness: Measurements were conducted using an ENS-TDV texture analyzer (Yinuoweiteng Technology Development Co., Ltd., Beijing, China) fitted with a P12 cylindrical probe. The trigger force was set to 0.1 N, with pre-test speed and post-test speed of 1 mm/s, a compression depth of 7 mm, and a deformation degree of 50%. For each cultivar, firmness was determined using 10 parallel measurements, and the results were expressed as the mean value [10].

The Folin–Ciocalteu method was adopted for total phenolic content. Different volumes of sweet cherry pulp homogenate supernatant were diluted with distilled water, followed by addition of Folin–Ciocalteu reagent and sodium carbonate solution; then the mixture was fixed to volume and incubated at 20 °C in the dark for 2 h. The absorbance value was detected at 765 nm with a spectrophotometer, and total phenol content was calculated based on gallic acid standard curve. Each sample was measured in triplicate, and the final result was expressed as the mean value [11].

Stalk length, fruit longitudinal diameter, and fruit transverse diameter were determined using electronic Vernier calipers (Guanglu Measuring Tools, Guilin, China). Fruit, stalk, and core weights were measured using an electronic analytical balance (Ohaus, Parsippany, NJ, USA). For each cultivar, all basic indicators were determined in 30 parallel replicates, and the results were expressed as mean values.

### 2.4. Color Parameters

Color determination: Four equidistant measurement points were uniformly marked on the equatorial region of a single sweet cherry. A CR-10 colorimeter (Konica Minolta, Tokyo, Japan) was used to determine three parameters under a D65 light source and a 10° viewing angle: L* (lightness/darkness), a* (redness/greenness), and b* (yellowness/blueness). Ten samples were randomly selected from each sweet cherry cultivar for color assessment. Each measurement was repeated three times, and the results were calculated as mean values [9].

### 2.5. Analysis of Organic Acids and Soluble Sugars

Samples were prepared according to the method described by Zhang et al. [12], with minor modifications. The 1260 high-performance liquid chromatograph (Agilent, Santa Clara, CA, USA) equipped with a Bio-Rad HPX-87H ion exclusion column (300 mm × 7.8 mm; Bio-Rad, Hercules, CA, USA) was used. The mobile phase was 0.01 mol/L sulfuric acid aqueous solution for isocratic elution. The injection volume was 5 μL, the flow rate was 0.6 mL·min^−1^, and the column temperature was maintained at 55 °C. A diode array detector (DAD) set at a wavelength of 210 nm was used to determine organic acids, while a refractive index detector (RID) was adopted for soluble sugar quantification.

### 2.6. Analysis of Phenolic Compositions

Samples were prepared according to the method described by Bian et al. [11], with minor modifications. The LC2030CD (Shimadzu, Kyoto, Japan) liquid chromatograph equipped with a Zorbax SB-C18 column (250 mm × 4.6 mm, 5 μm; Agilent) was used. Mobile phase A consisted of a 1% formic acid aqueous solution, and mobile phase B consisted of pure acetonitrile. The injection volume was 10 μL, the flow rate was 0.95 mL·min^−1^, and the detection wavelength was 280 nm. The column temperature was maintained at 25 °C.

### 2.7. Analysis of Volatile Compounds

Aromatic compounds in sweet cherry samples were extracted using headspace solid-phase microextraction (HS-SPME) and analyzed using gas chromatography–mass spectrometry (GC-MS), following the method outlined by Zhang et al. [13].

Extraction of aroma compounds from sweet cherry samples: According to biological replicates, 50 g of each replicate was sampled, flash-frozen in liquid nitrogen, immediately removed from the stems, crushed, and the seeds were removed under liquid nitrogen protection; the remaining fruit parts were mixed with D-gluconolactone (0.5 g) and PVPP (2 g) and ground into powder using a grinder. The sweet cherry powder was mixed thoroughly, followed by static extraction at 4 °C for a minimum duration of 4 h, centrifuged at 8000× *g* for 10 min at 4 °C, and then the supernatant was collected. Next, NaCl (1 g) and the supernatant (5 mL) were accurately weighed and transferred into a 15 mL sample vial, followed by the addition of a 1 cm magnetic stir bar. Subsequently, 10 μL of the internal standard, 4-methyl-2-pentanol (4M2P, 1.0083 g/L), was pipetted into the vial, which was immediately sealed with a polytetrafluoroethylene (PTFE)-lined heat-insulating cap. Prior to extraction, a 50/30-μm DVB/Carboxen/PDMS extraction fiber (Supelco, Bellefonte, PA, USA) was conditioned in the GC injection port at 250 °C for 30 min to remove residual contaminants. The sample vials were placed on a magnetic heating stirrer and equilibrated at 40 °C for 30 min, and then gently stirred. The preconditioned extraction fiber was inserted into the headspace and extracted at 40 °C for 30 min. After extraction, the fiber was withdrawn and immediately inserted into the GC injection port for desorption at 250 °C for 8 min.

Determination of aroma compounds: Aromatic compound separation and identification were performed using an Agilent 6890 GC coupled with an Agilent 5975 mass spectrometer (MS). The GC column was a 60 m × 0.25 mm HP-INNOWAX capillary column with a 0.25 μm film thickness (J & W Scientific, Folsom, CA, USA). Helium (purity > 99.999%) was used as the carrier gas at a flow rate of 1 mL/min. The initial oven temperature was set to 50 °C, maintained for 1 min, then increased to 220 °C at a rate of 3 °C/min, and maintained for 5 min. The mass spectrometer was operated in the electron ionization (EI) mode at 70 eV, initially performing a full-scan (SCAN) mode with a mass scan range of *m*/*z* 30–350 for the qualitative screening and identification of volatile aroma compounds; the ion source temperature was set to 230 °C, and the MS transfer line temperature was maintained at 280 °C. Subsequently, the selected ion monitoring (SIM) mode was applied for the quantitative determination of the concentrations of aroma compounds and the internal standard 4-methyl-2-pentanol, and the characteristic *m*/*z* ions of each target compound were selected for monitoring.

Qualitative and quantitative analyses: Volatile compounds were qualitatively identified by matching the mass spectral information obtained from the SCAN mode with the NIST database, and by comparing the experimentally determined retention indices with those in the NIST database and/or authentic standards. Peak integration was performed using the MSD ChemStation software (version E.02.02, Agilent Technologies, Santa Clara, CA, USA). Standard curves were constructed using known concentrations (μg/L) of authentic standards to quantify volatile compounds. Briefly, calibration curves for aroma compounds were established based on mixed standard solutions serially diluted to 15 concentration gradients (μg/L) in a synthetic model matrix, with 4-methyl-2-pentanol as the internal standard. The linearity of the calibration curves was assessed by evaluating heteroscedasticity and inspecting outliers and regression parameters according to the methods described by Gu et al. [14]. Compounds lacking calibration standards were quantified based on similar carbon chain lengths and/or functional groups.

### 2.8. Statistical Analysis

All experiments were performed with three independent biological replicates. Each technical analysis was performed in triplicate. The results are expressed as the mean ± standard deviation (SD). Statistical analyses were performed using SPSS 26.0 (SPSS Inc., Chicago, IL, USA) with data from three replicates. Analysis of variance (ANOVA) was used to assess the significance of the differences among samples, with the significance level set at *p* < 0.05, followed by Tukey’s HSD test. Graphs were created using OriginPro 2021 software (OriginLab Corporation, Northampton, MA, USA).

## 3. Results and Discussion

### 3.1. Physicochemical Properties

#### 3.1.1. Basic Physicochemical Properties

**Figure 1:**
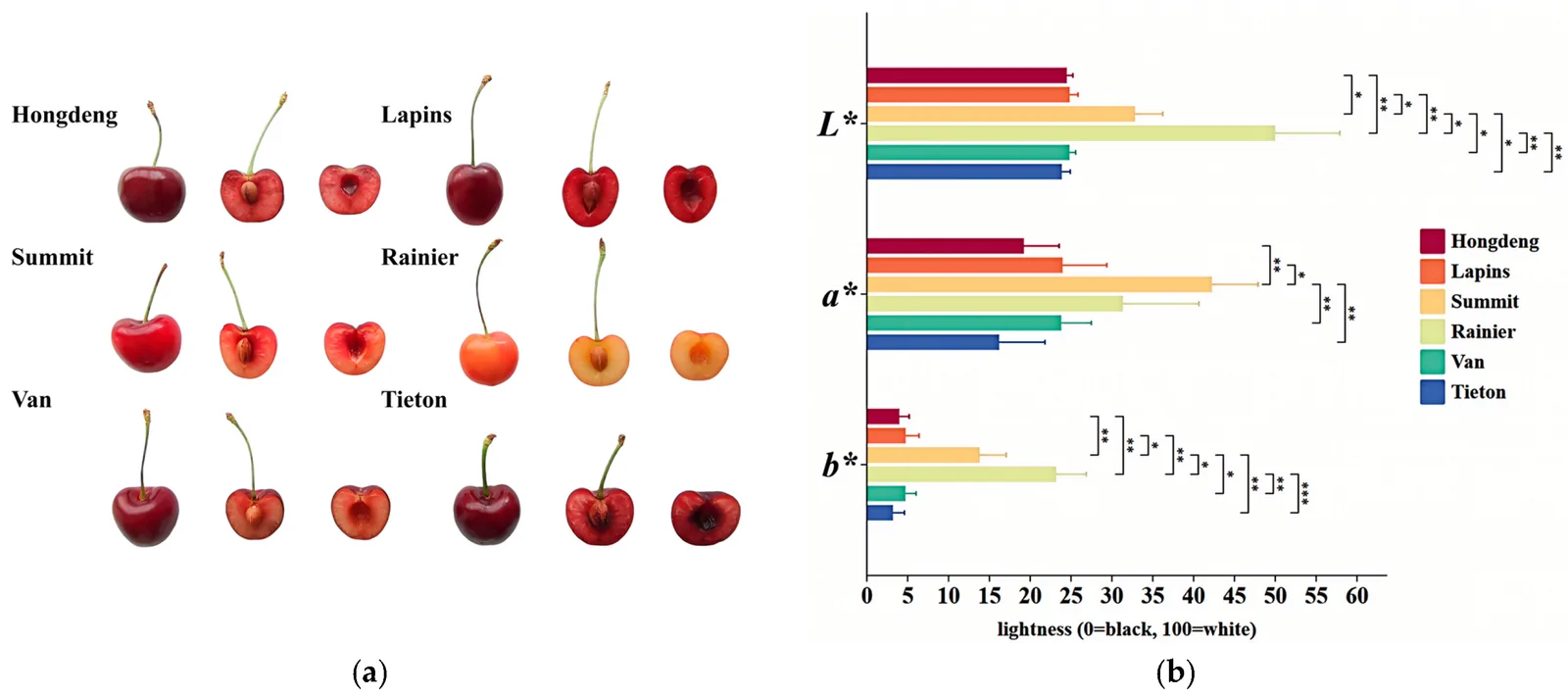
Basic physicochemical properties of different sweet cherry cultivars. (**a**) Fruit morphology. (**b**) Color parameters. * *p* < 0.05, ** *p* < 0.01,*** *p* < 0.001.

**Figure 2:**
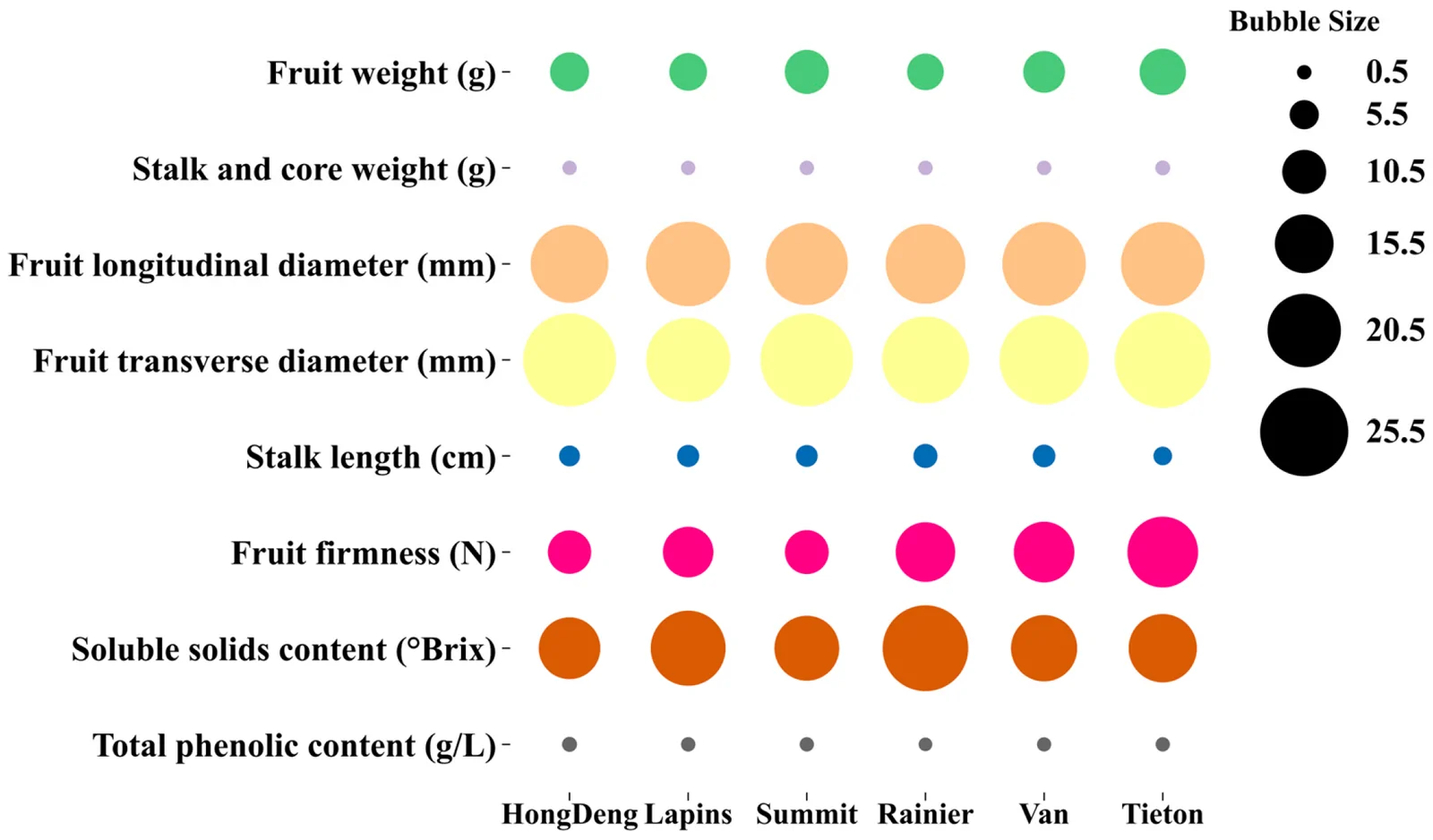
Basic physicochemical properties of different sweet cherry cultivars.

As shown in Figure 1 and Figure 2 and Table S2, the six sweet cherry cultivars differed significantly in their major quality attributes, including fruit morphology, texture, sweetness, total phenolic content, and skin color. Tieton had the largest transverse diameter at 28.11 mm and the greatest single-fruit weight at 11.37 g, followed by Summit at 27.00 mm and 10.59 g, respectively. Rainier exhibited the smallest values for both parameters, with a transverse diameter of 25.01 mm and a single-fruit weight of 8.08 g. The longitudinal diameter ranged from 21.97 mm in Hongdeng to 24.23 mm in Lapins. Rainier had the longest stalk at 3.80 mm, whereas Tieton had the shortest at 1.95 mm. The combined weight of the stalk and core ranged from 0.47 g in Lapins to 0.67 g in Tieton. Rainier had the highest soluble solids content at 24.67 °Brix, substantially exceeding the values measured in the other cultivars, followed by Lapins at 21.03 °Brix. Hongdeng had the lowest soluble solids content at 16.53 °Brix. Soluble solids content is a key indicator of sweetness and directly affects consumer preference. The relatively high values observed in most cultivars may be associated with the arid climate of Kashgar, which is characterized by intense solar radiation, large diurnal temperature fluctuations, and low precipitation during fruit development. These conditions can promote sugar accumulation. Tieton and Van had the greatest firmness values at 19.57 N and 16.14 N, respectively, whereas Hongdeng and Summit had relatively soft flesh, with firmness values of 10.31 N and 10.47 N, respectively. Firmness is an important quality attribute for postharvest handling and marketability. The firmer cultivars, Tieton and Van, are more suitable for long-distance transportation and extended storage, whereas the softer cultivars, Hongdeng and Summit, may be more appropriate for local fresh consumption. Total phenolic content also varied markedly among cultivars. Hongdeng had the highest value at 0.69 g/L, followed by Lapins at 0.50 g/L, while Rainier had the lowest concentration at 0.25 g/L. This variation may be related to differences in peel pigmentation because red-skinned cherries generally accumulate higher levels of phenolic antioxidants than yellow-blush cultivars. With respect to skin color, Summit had the highest a* value at 42.23, indicating the most intense red coloration and corresponding to the preference for dark-red cherries in the premium fresh-fruit market. Rainier had the highest L* and b* values at 49.97 and 23.14, respectively, confirming its characteristic yellowish-red peel. The diversity in skin color provides opportunities for market differentiation, with dark-red cultivars such as Summit and Lapins being suitable for mainstream premium markets and the yellowish-red Rainier having potential in high-end gift and specialty-fruit markets.

#### 3.1.2. Organic Acids, Soluble Sugars and Individual Phenolic Compounds

**Figure 3:**
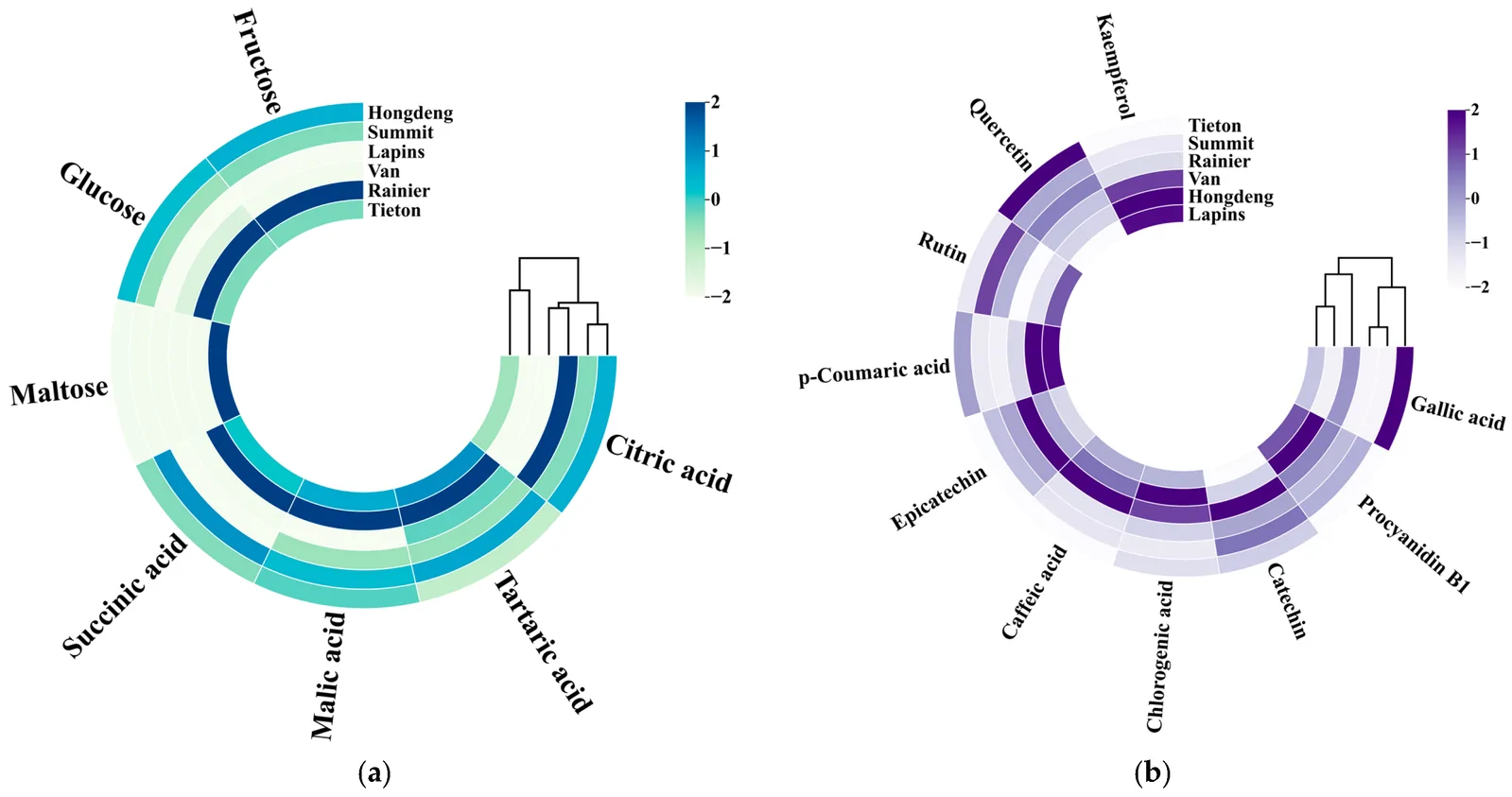
Circular heatmaps of organic acids, soluble sugar and individual phenolic compounds in six sweet cherry cultivars. (**a**) Organic acids and soluble sugars. (**b**) Individual phenolic compound profiles. The color scale represents normalized values ranging from −2 to 2, with the dendrogram illustrating hierarchical clustering of cultivars based on their metabolite accumulation patterns.

The organic acid and soluble sugar contents in the juice of the six sweet cherry cultivars are presented in Figure 3a and Table S3. Malic acid was the predominant organic acid, with concentrations ranging from 11.56 to 20.27 g/L, followed by citric acid at 5.30 to 14.18 g/L. Tartaric acid and succinic acid were detected at relatively low concentrations in all cultivars. The soluble sugar fraction was dominated by glucose and fructose, which ranged from 47.32 to 84.14 g/L and from 46.97 to 78.39 g/L, respectively. Maltose was detected only at trace levels, ranging from 0.06 to 0.08 g/L. Marked cultivar-specific patterns of organic acid accumulation were observed. The contrasting acid profiles of ‘Rainier’, which had a high malic acid content and a low citric acid content, and ‘Lapins’, which showed the opposite pattern, provided a clear example. These differences indicate that organic acid metabolism is subject to genetic regulation and contributes to the variation in flavor characteristics among sweet cherry cultivars. In addition to modulating sensory perception, organic acids may influence the nutritional quality of sweet cherry fruit. The acidic microenvironment maintained by organic acids may interact with endogenous antioxidants, including phenolic compounds and ascorbic acid, thereby contributing to the overall antioxidant capacity of the fruit [15]. This finding highlights the potential physiological functions of organic acids beyond their role in flavor regulation.

The concentrations of ten individual phenolic compounds in the six sweet cherry cultivars are shown in Figure 3b and Table S3. Among the phenolic acids, p-coumaric acid and chlorogenic acid were the most abundant, with concentration ranges of 30.03 to 68.72 mg/L and 27.25 to 44.32 mg/L, respectively. Rutin was the predominant flavonol, with concentrations ranging from 25.85 to 35.18 mg/L. This result is consistent with previous reports that hydroxycinnamic acids and flavonols constitute the major phenolic fractions in sweet cherry fruit [16]. Distinct cultivar-dependent patterns of phenolic accumulation were observed among the tested cultivars. ‘Hongdeng’ had a markedly higher procyanidin B1 concentration at 77.46 mg/L than the other five cultivars. ‘Van’ exhibited the highest caffeic acid content at 0.91 mg/L, whereas ‘Tieton’ accumulated relatively high levels of gallic acid and quercetin at 2.48 and 4.10 mg/L, respectively. ‘Rainier’, a yellow-blush cultivar, had a relatively low epicatechin concentration of 3.17 mg/L, which is consistent with the predominant accumulation of phenolic metabolites in fruit peel. The cultivar-dependent variation in phenolic profiles may be associated with genotypic differences and variation in the activities of key enzymes involved in phenolic biosynthesis.

### 3.2. Volatile Compounds of Different Cultivars of Sweet Cherries

Gas chromatography–mass spectrometry (GC-MS) was employed for the qualitative and quantitative analysis of aroma components in sweet cherries [17]. A total of 56 aroma compounds were detected, including 18 esters, 20 alcohols, four aldehydes, seven acids, one phenol, three terpenes, and four other compounds. In sweet cherries, the total concentration of aroma compounds in the Rainier cultivar was 67,407.18 ± 4139.09 μg/L, which was the highest total concentration among all cultivars. The total aroma concentrations in Van and Tieton were higher than those in Hongdeng, whereas the Lapins had the lowest total aroma concentration. Alcohols accounted for the highest proportion of aroma compounds in all sweet cherry cultivars, whereas phenols and terpenes accounted for the lowest proportion, indicating that alcohols were the main contributors to the aroma of the six sweet cherry cultivars. This finding is consistent with previous reports on the volatile composition of sweet cherry. Vavoura et al. [8] found that alcohols were the predominant volatile compounds in four Greek sweet cherry cultivars, accounting for more than 70.00% of the total aroma compounds. Similarly, Villavicencio et al. [7] identified alcohols as the major aroma constituents in ‘Regina’ sweet cherries cultivated under temperate conditions. In the present study, alcohols accounted for 74.94% to 84.63% of the total volatile compounds, a proportion slightly higher than that reported for cherries produced in temperate humid regions. This difference may be associated with the arid environmental conditions of Kashgar, Xinjiang. Intense solar radiation and large diurnal temperature variations may promote the biosynthesis and accumulation of alcohol-derived volatiles in sweet cherry flesh.

#### 3.2.1. Esters

**Figure 4:**
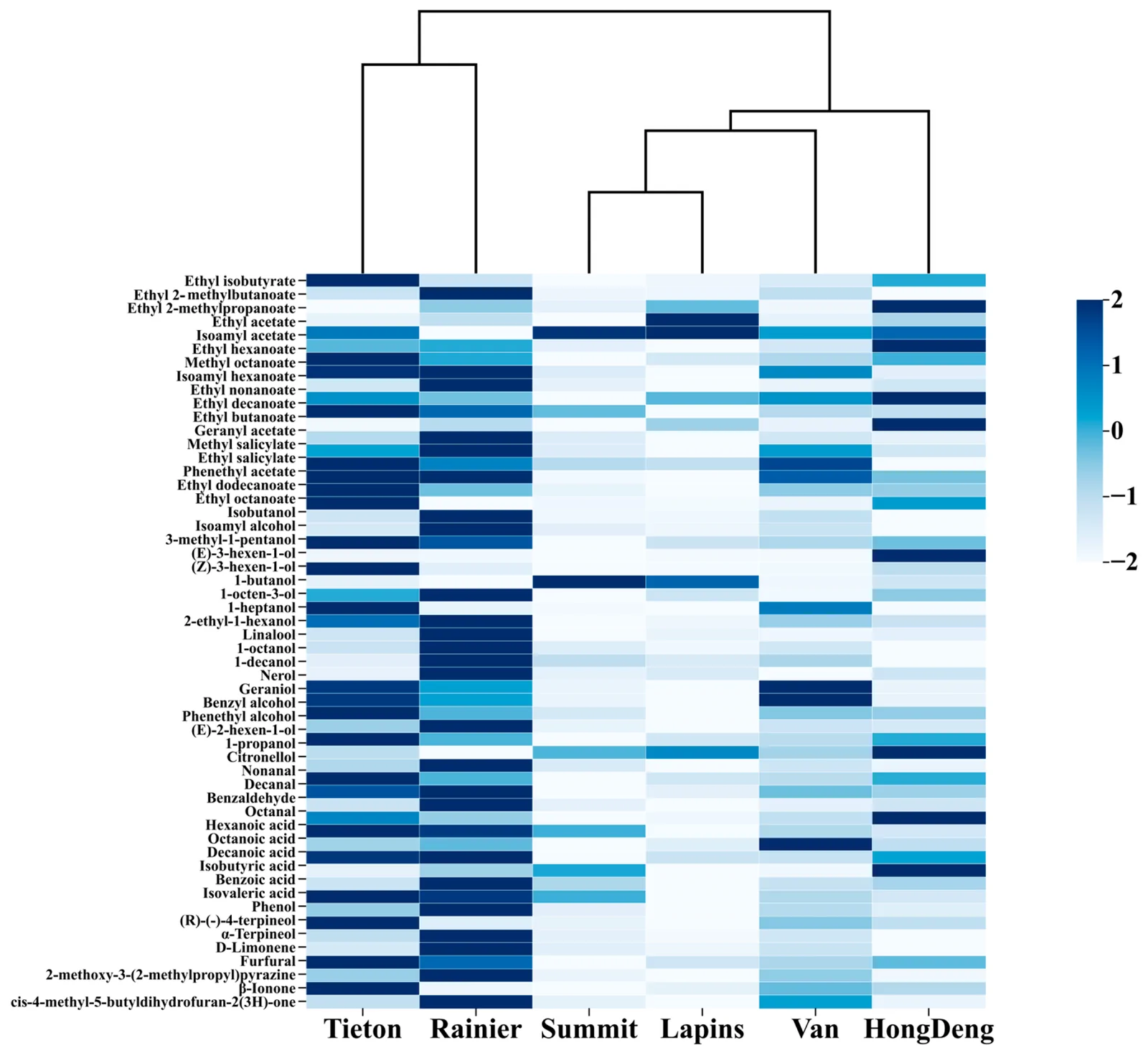
Volatile aroma compounds in different sweet cherry cultivars. Heatmap of volatile compound concentrations. The color scale represents normalized values, ranging from −2 (low aroma intensity/contribution, light blue) to 2 (high aroma intensity/contribution, dark blue), with the dendrogram illustrating hierarchical clustering of samples based on their aroma composition.

Esters are typically volatile and odoriferous, contributing substantially to the aroma of sweet cherry and functioning as a key determinant of its taste and flavor. An appropriate concentration of esters can enhance the quality and appeal of sweet cherries; however, excessive concentrations may disrupt the balance between taste and flavor [18]. Esters constituted one of the main groups of volatile compounds in the six sweet cherry cultivars, with total concentrations varying significantly among the cultivars (Figure 4 and Table S4). Total ester concentrations among the six sweet cherry cultivars ranged from 966.47 μg/L in ‘Summit’ to 1299.42 μg/L in ‘Rainier’. ‘Rainier’ exhibited the highest total ester content, followed by ‘Lapins’ at 1280.14 μg/L. The ester concentrations in ‘Hongdeng’, ‘Van’, and ‘Tieton’ were 1054.86, 1037.91, and 1029.19 μg/L, respectively. ‘Summit’ had the lowest ester concentration. Esters represented 1.93% to 7.41% of the total volatile compound pool across the tested cultivars. These compounds are key contributors to the fruity and floral sweet cherry aroma [18]. Ethyl butanoate and ethyl acetate are the most abundant esters in sweet cherries, and are key contributors to the fruity (apple and banana) aroma at optimal concentrations [19]. We observed that the ethyl 2-methylbutanoate content varied substantially among different sweet cherry cultivars, which is strongly associated with differences in the fruity aroma intensity of different cultivars. This indicates that the type and content of esters directly affect the aromatic characteristics of sweet cherries, thereby determining the unique flavor of each cultivar. Notably, the Rainier cultivar had the highest ethyl 2-methylbutanoate content, which may be the main reason for its unique sweet and fruity aroma. This difference in ester accumulation among cultivars is consistent with previous observations [20], and may be attributed to differences in the activity of ester-synthesizing enzymes in different sweet cherry cultivars or differential substrate availability. The relatively high ester content in ‘Lapins’ is in agreement with the cultivar-specific flavor characteristics reported by Vavoura et al. [8]. However, esters accounted for a slightly lower proportion of the total volatile compounds in the present samples than in cherries grown in humid temperate regions. This variation may be related to the arid climate of Kashgar, Xinjiang, where intense solar radiation and large diurnal temperature fluctuations may favor alcohol biosynthesis over ester formation.

#### 3.2.2. Volatile Alcohols

Volatile alcohols, the key category of aroma compounds in the six sweet cherry cultivars, notably affect the aroma characteristics of sweet cherries because of their content, composition, and the differences between cultivars. Based on the data in Table S4 and Figure 4, the alcoholic aroma compounds in all six sweet cherries included 20 specific components, with no differences in the number of types, indicating that the compositional diversity of this class of compounds was stable among the tested sweet cherry cultivars, with no components missing or newly added because of differences in cultivars.

Volatile alcohols dominated the aroma profile, with total concentrations ranging from 13,767.25 μg/L in ‘Lapins’ to 54,846.05 μg/L in ‘Rainier’. The total alcohol concentrations in ‘Tieton’ and ‘Van’ were 24,840.09 and 22,543.57 μg/L, respectively. Alcohols accounted for 74.94% to 84.63% of the total volatile compounds across all tested cultivars. Notably, the total amount of alcohols in Rainier was approximately four times that in Lapins, indicating that variety characteristics have a significant regulatory effect on the overall accumulation of alcohols.

Regarding key components, isobutanol, isoamyl alcohol, 1-butanol, phenethyl alcohol, and 1-propanol were the main contributors among the alcohols, with their contents notably exceeding those of the other components. Specifically, notably high concentrations of isobutanol and isoamyl alcohol were observed in Rainier, which were more than six times those of Summit and Lapins. This is the primary reason for the substantially increased total alcohol content in Rainier. In addition, benzyl alcohol showed significant differences among the different cultivars, with Tieton exhibiting the highest content and Lapins the lowest concentration, thereby becoming one of the key indicators for distinguishing the alcohol component characteristics of the different cultivars. ‘Rainier’ exhibited notably high levels of isobutanol and isoamyl alcohol, in agreement with the flavor profile previously reported for this yellow-fleshed cultivar by Sun et al. [20]. The benzyl alcohol content of ‘Tieton’ was substantially higher than that reported for cherry orchards in eastern China, indicating that regional climatic conditions may regulate the biosynthesis of aromatic alcohols. Regarding component details, the levels of certain alcohol compounds were relatively consistent among different cultivars, such as 1-octen-3-ol, 1-decanol, and citronellol, with minimal fluctuations in their content. This indicates that the accumulation of these compounds is less affected by variety and may be considered the basic components of alcohol-type aroma compounds in sweet cherries. In contrast, unsaturated alcohol compounds, such as (E)-3-hexen-1-ol and (Z)-3-hexen-1-ol, demonstrated substantial differences in content. The (E)-3-hexen-1-ol content in Hongdeng was much higher than that in the other cultivars, whereas the (Z)-3-hexen-1-ol content in Tieton was significantly higher than that of similar cultivars. These compounds may be related to the fresh herbal aroma of sweet cherries, and differences in their contents may result in subtle differences in the aroma and flavor of sweet cherries from different cultivars [21].

#### 3.2.3. Acids

Acids, essential aroma and taste-active compounds in sweet cherry fruits, influence the unique sour-sweet flavor characteristics of different sweet cherry cultivars and are involved in the formation of other aroma substances, such as esters, through esterification reactions, thereby affecting the overall sensory quality of sweet cherries [21]. Based on the data in Figure 4 and Table S4, the acid category in the six sweet cherry cultivars consisted of seven specific compounds, with no difference in the number of components among cultivars, indicating that the component composition of acidic aroma compounds was relatively conserved in the tested sweet cherry cultivars.

Significant differences were observed in the accumulation of acidic compounds among different sweet cherry cultivars. Total volatile acid concentrations ranged from 751.21 μg/L in ‘Lapins’ to 1128.70 μg/L in ‘Rainier’. ‘Rainier’ exhibited the highest total acid content, whereas ‘Lapins’ had the lowest. From the perspective of proportion in the total aroma compounds, the proportion of the acids category in each sweet cherry variety was relatively moderate, ranging from 1.67% (Rainier) to 4.62% (Summit). Summit had the highest proportion of acids, followed by Hongdeng (4.36%) and Lapins (4.35%), and Rainier had the lowest proportion of acids (only 1.67%), which is related to its significantly higher total aroma compounds compared to those of other cultivars. Although the proportion of acids was lower than that of alcohols (74.94–84.63%), it was higher than that of phenols and terpenes, indicating that acids are important auxiliary components in the sweet cherry aroma system, and their content and proportion directly affect the coordination of the sweet cherry flavor [19]. Notably, the total acid content of Rainier was approximately 1.5 times that of Lapins, indicating that the genetic characteristics of sweet cherry cultivars have a pronounced regulatory effect on the accumulation of acidic aroma compounds [22].

In terms of key component distribution, hexanoic, octanoic, isovaleric, isobutyric, and benzoic acids were the main components of the acids category, and their content accounted for a substantial proportion of the total acid content. Hexanoic acid content exhibited the most significant differences among the cultivars. Among all cultivars, Rainier and Tieton had significantly higher values in hexanoic acid content, whereas Lapins had the lowest concentration, with a difference of approximately 1.4 times compared to that of Tieton. Octanoic and decanoic acid contents were relatively consistent across different cultivars, with minimal fluctuations, indicating that these two acidic compounds are the basic components of sweet cherry acids and are less affected by varietal differences. Hexanoic acid and octanoic acid were identified as the predominant acidic volatile compounds in the present study, consistent with previous reports [23].

#### 3.2.4. Terpenes

Terpenes are important characteristic aroma compounds in sweet cherry fruits, primarily produced by the metabolic processes of sweet cherry berries, and their types and concentrations are closely related to the genetic background of sweet cherry cultivars, which directly affects the unique aromatic characteristics of different sweet cherry cultivars [5]. These compounds provide sweet cherries with distinct floral, citrus, and woody notes as well as contribute to the formation of the overall aroma system of sweet cherries, thereby becoming key indicators for evaluating the sensory quality and variety characteristics of sweet cherries [5]. Terpenoids occurred at trace levels in all tested sweet cherry cultivars. The total terpene concentration was highest in ‘Rainier’ at 67.78 μg/L and lowest in ‘Hongdeng’ at 15.33 μg/L, whereas ‘Lapins’ contained 17.15 μg/L. Three terpenoid compounds were quantified across all cultivars, and the total terpene fraction accounted for approximately 0.10%. This is related to the inherent metabolic characteristics of terpene compounds; although their content is low, they have strong volatility and aroma activity. Trace amounts of terpenes can notably affect the aromatic characteristics of sweet cherries, providing different cultivars with unique flavor labels [24]. This finding is consistent with the trace terpene feature of sweet cherries summarized by Wen et al. [23]. Based on the data in Figure 4, the terpenes category in six sweet cherry cultivars comprised three specific compounds, namely (R)-(-)-4-terpineol, α-Terpineol, and D-Limonene, with no difference in the number of components among cultivars, indicating that the component composition of terpenes is relatively consistent in the tested sweet cherry cultivars. Terpenoids such as R-(-)-4-terpineol are bioactive compounds with potential anti-inflammatory activities in model systems [25]. Although terpenoids accounted for only approximately 0.10% of the total volatile compounds in the present study, their low odor thresholds may enable them to contribute subtle floral and citrus notes at trace concentrations, thereby increasing aroma complexity. Among the six cultivars, ‘Rainier’ had the highest total terpenoid content, which may have contributed to its more complex aroma profile. Further studies are needed to clarify the bioactivity and dietary relevance of these trace terpenoids in sweet cherries.

#### 3.2.5. Aldehydes

Aldehydes are important volatile aroma compounds in sweet cherry fruits, mainly formed by the oxidation and degradation of fatty acids and terpenoids in sweet cherry berries during growth and postharvest storage [26]. Their unique aromatic properties and concentration characteristics are closely related to the variety and maturity of sweet cherries, which provide sweet cherries with fresh, fruity, and floral notes and are critical in enhancing the complexity of the sweet cherry aroma. Based on the data in Figure 4 and Table S4, the aldehyde category in the six sweet cherry cultivars consisted of four specific compounds, nonanal, decanal, benzaldehyde, and octanal, with no difference in the number of components among the cultivars, indicating that the component composition of aldehydes was relatively consistent in the tested sweet cherry cultivars.

Total aldehyde concentrations ranged from 1384.05 μg/L in ‘Lapins’ to 9944.18 μg/L in ‘Rainier’. ‘Rainier’ exhibited the highest aldehyde content, which was more than seven times that detected in ‘Lapins’. ‘Tieton’ and ‘Hongdeng’ also showed relatively high aldehyde concentrations, at 3093.10 and 2909.08 μg/L, respectively. Aldehydes accounted for 7.86% to 14.78% of the total volatile compounds across the sweet cherry cultivars. In terms of the key component distribution, benzaldehyde was the dominant aldehyde component in cherries, and its content accounted for an absolute proportion of the total aldehyde content, showing the most significant difference among cultivars. Rainier had the highest content of benzaldehyde, which was substantially higher than that of the other cultivars; Tieton and Hongdeng had the second highest content; Van and Summit had moderate contents; and Lapins had the lowest content of benzaldehyde. The significant difference in benzaldehyde content was the key factor resulting in the difference in total aldehyde content among cultivars, because benzaldehyde is an important source of almond, sweet cherry, and floral aroma in sweet cherries, and its content directly determines the intensity and characteristic flavor of the aldehyde-derived aroma in sweet cherries [23]. The significant variation in benzaldehyde concentration among cultivars observed in the present study is consistent with the cultivar-dependent differences reported by Sun et al. [20]. Compared with cherries cultivated in low-altitude humid regions, the samples analyzed here contained markedly higher benzaldehyde levels, which may be associated with the high solar radiation in the arid environment of Kashgar and its potential stimulation of the phenylpropanoid pathway. Benzaldehyde has been reported to exhibit antioxidant activity in certain in vitro model systems, although the functional significance of its trace-level presence in cherries consumed as part of the diet remains unclear [27]. The pronounced differences in benzaldehyde content among the six sweet cherry cultivars may contribute to cultivar-dependent sensory characteristics and provide a potential basis for differentiating their bioactive compound profiles.

### 3.3. Odor Activity Value Analysis

The odor activity value (OAV) is a pivotal quantitative metric for evaluating the contribution of individual volatile compounds to the characteristic aroma of different sweet cherry cultivars, thereby enabling direct comparison of their sensory relevance across genotypes [28]. By integrating GC-MS-derived volatile concentrations with published odor thresholds, OAVs were calculated for all detected compounds across the six cultivars, revealing distinct patterns of aroma-active components that underpin cultivar-specific flavor profiles.

**Figure 5:**
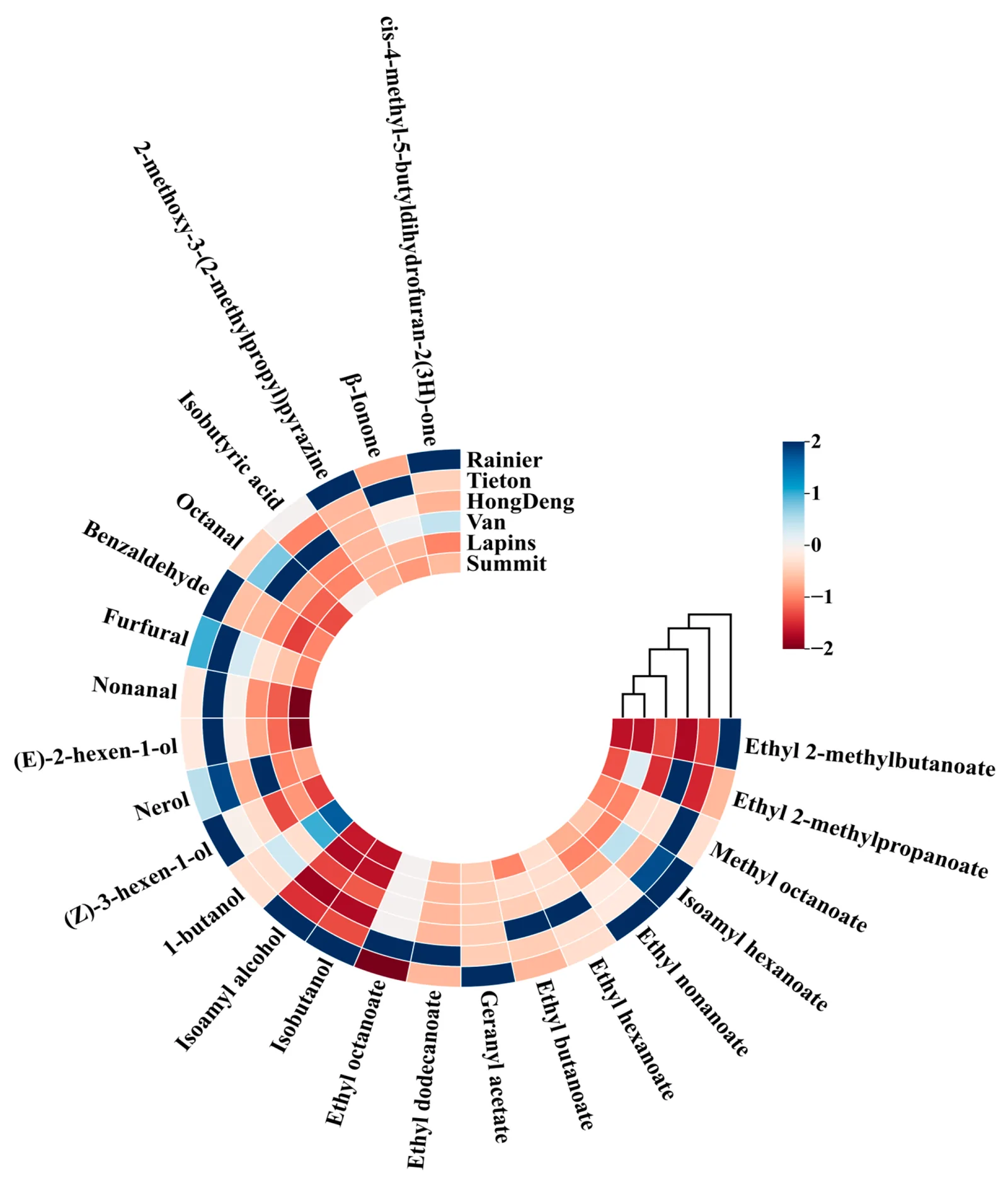
Volatile aroma compounds in different sweet cherry cultivars. The color scale represents normalized OAV values, ranging from −2 (low aroma intensity/contribution, light blue) to 2 (high aroma intensity/contribution, dark blue), with the dendrogram illustrating hierarchical clustering of samples based on their aroma composition.

Among the aroma compounds with an OAV > 1, three compounds exhibited an OAV exceeding 100, including two esters, ethyl 2-methylpropanoate and ethyl octanoate, which both demonstrated typical fruity characteristics (Table S5 and Figure 5). Additionally, one alcohol, (E)-2-hexen-1-ol, characterized by both fresh herbal and fruity notes, and another compound, β-Ionone, with a unique floral aroma, collectively formed the core aroma components of sweet cherry.

Notably, β-ionone predominantly exhibited the highest OAV, driven by its extremely low odor threshold, indicating its dominant role in the overall aroma of all cultivars, regardless of minor concentration variations. (E)-2-hexen-1-ol, a key C6 aldehyde-derived alcohol, also exhibited notably high OAV values, highlighting its contribution to the fresh green notes that are characteristic of sweet cherry fruits. Esters, particularly ethyl dodecanoate, ethyl octanoate, and ethyl 2-methylpropanoate, consistently ranked among the top aroma-active compounds, with OAVs exceeding 1000, underscoring their critical role in the fruity and sweet aromatic notes that define sweet cherry flavor. In contrast, compounds such as isobutanol and isoamyl alcohol, which were present at higher absolute concentrations, exhibited lower OAVs owing to their higher odor thresholds, with only Rainier showing significantly elevated OAVs among these higher alcohols.

### 3.4. Identification of Key Aroma Compounds

**Figure 6:**
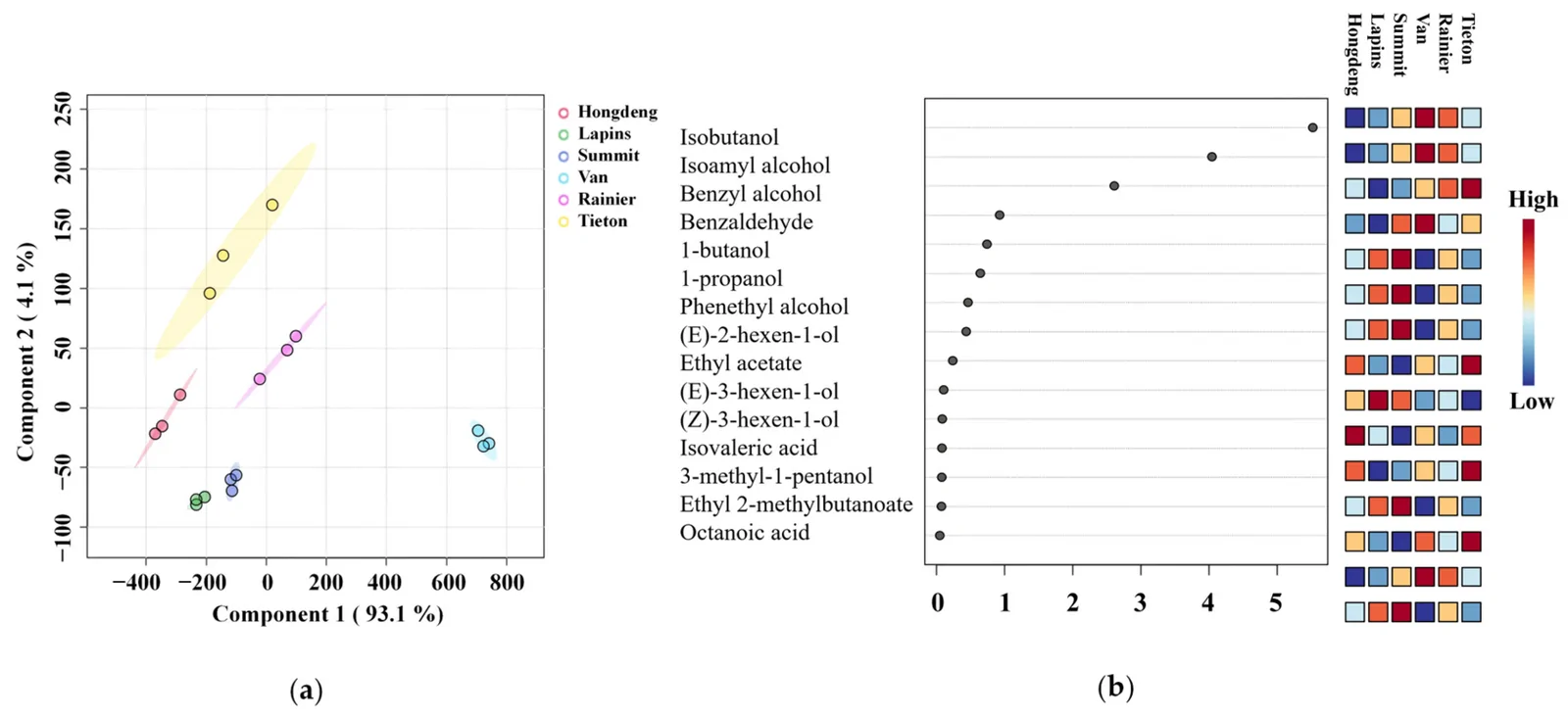
Multivariate discrimination of different sweet cherry cultivars. (**a**) PCA score plot; (**b**) PLS-DA score plot.

As shown in Figure 6a, based on the volatile profiles of the six sweet cherry cultivars, the first two principal components of principal component analysis (PCA) explained 97.2% of the total variance and exhibited distinct variety-specific clustering patterns. The six cultivars were categorized into four distinct groups: (i) Tieton is located in the positive direction of PC2 and is closely associated with high contents of compounds such as isobutanol, isoamyl alcohol, and benzaldehyde; (ii) Rainier forms a separate group, located in the region of positive PC1 and medium-high PC2 values; (iii) Hongdeng, Lapins, and Summit cluster in the negative PC1 region, with Hongdeng in the medium PC2 area, and Lapins and Summit in the low PC2 area; (iv) Van is located at the most positive end of PC1 and is associated with high contents of ester and acid compounds such as octanoic acid, ethyl 2-methylbutanoate, and isovaleric acid. This distinct distribution indicates that each variety exhibits unique volatile characteristics that are determined by the specific abundance of key aroma compounds.

Partial least squares discriminant analysis (PLS-DA) was conducted to identify the compounds influencing these cultivar differences. The VIP plot in Figure 6b shows the most influential volatile compounds responsible for the separation. Key discriminant markers included higher alcohols (isobutanol and isoamyl alcohol), aromatic alcohols (benzyl alcohol and phenethyl alcohol), and aldehydes (benzaldehyde). These compounds contribute to diverse sensory attributes; isobutanol and isoamyl alcohol provide alcoholic and fermentative notes, benzaldehyde contributes almond-like nuances, and esters impart fruity characteristics [23]. The cherry aroma is closely related to two key metabolic pathways. Unsaturated fatty acids are catalyzed by lipoxygenase (LOX) and hydroperoxide lyase (HPL) to form C6 aldehydes and alcohols, such as (E)-2-hexen-1-ol, which are the primary sources of green and fresh notes [29]. Additionally, alcohol acyltransferase (AAT) catalyzes the esterification of alcohols and acyl-CoA to produce ethyl 2-methylbutanoate, ethyl octanoate, and other esters, which are responsible for the strong fruity aroma [30].

The variation in volatile components among the sweet cherry cultivars can be attributed to the different activities of LOX, HPL, and AAT during fruit ripening. Consequently, the abundance of these compounds varies by cultivar, directly determining the unique flavor profile of each sweet cherry cultivar.

### 3.5. Pearson Correlation Analysis

To elucidate the relationship between fruit quality and characteristic flavor, Pearson correlation analysis was performed between basic physicochemical indicators and key aroma-active compounds, which were strictly screened according to the following criteria: (1) odor activity value (OAV) > 1, representing actual contribution to cherry aroma; (2) identified as important discriminant markers by PCA and PLS-DA (VIP > 1); (3) significant differences among cultivars (*p* < 0.05); and (4) covering four major aroma categories including green, fruity, floral, and almond-like notes. Finally, eight key aroma compounds were included in the correlation analysis to ensure the accuracy and representativeness of the results.

As presented in Table 1, the Pearson correlation coefficient matrix between the basic physicochemical indicators of the six sweet cherry cultivars and the key aroma-active compounds with OAV > 1 showed that isobutanol, isoamyl alcohol, and benzaldehyde were significantly positively correlated with soluble solids content, peel lightness L*, yellowness b*, and pedicel length, with the highest correlation coefficient of 0.88. These are the key aromatic compounds that determine the intrinsic flavor and appearance of sweet cherries. Benzyl alcohol was significantly positively correlated with fruit transverse diameter, single fruit weight, pedicel core weight, and fruit hardness, and can be used as a core aroma marker for sweet cherry fruit quality. (E)-2-hexen-1-ol was significantly and positively correlated with fruit hardness, whereas ethyl 2-methylpropanoate was significantly and negatively correlated with single-fruit weight, fruit hardness, and other indicators. The two aroma compounds β-ionone and ethyl caprylate, as well as the peel redness a* indicator, showed no significant linear correlation with any of the corresponding indicators. Because all cultivars were evaluated under the same orchard and climatic conditions, the observed differences primarily reflected cultivar-related variation. Nevertheless, the regional arid climate may have influenced the overall accumulation of sugars, organic acids, phenolics, and volatile compounds. Multi-location and controlled-environment studies are therefore warranted to further disentangle genotype, environmental, and genotype × environment effects.

## 4. Conclusions

This study comprehensively characterized the fruit quality, sugar and organic acid composition, phenolic profiles, and aroma characteristics of six sweet cherry cultivars grown in the arid region of Mixia Town, Shache County. Significant cultivar-dependent differences were observed in fruit weight, transverse diameter, firmness, soluble solids content, total phenolic content, and skin color. Glucose and fructose were the predominant sugars, whereas malic acid and citric acid were the major organic acids. ‘Rainier’ contained more malic acid but less citric acid, while ‘Lapins’ showed the opposite pattern. The predominant phenolic acids were p-coumaric acid and chlorogenic acid, and rutin was the major flavonol. ‘Hongdeng’ had the highest procyanidin B1 and total phenolic contents, whereas ‘Rainier’ had the lowest phenolic concentration. A total of 56 volatile compounds were identified, with alcohols accounting for 74.94–84.63% of the volatile profiles. ‘Rainier’ showed the highest volatile abundance and soluble solids content, as well as the highest L* and b* values, and was mainly characterized by isobutanol, isoamyl alcohol, and benzaldehyde. ‘Van’ contained relatively high levels of octanoic acid and ethyl 2-methylbutanoate, whereas ‘Tieton’ had the largest fruit size, greatest firmness, and highest benzyl alcohol content. ‘Hongdeng’ exhibited relatively low firmness and was characterized by E-3-hexen-1-ol. β-Ionone, E-2-hexen-1-ol, ethyl 2-methylpropanoate, and ethyl octanoate were identified as core aroma compounds, with odor activity values exceeding 1000. PCA and PLS-DA further identified isobutanol, isoamyl alcohol, benzaldehyde, and benzyl alcohol as important discriminant markers, with VIP values above 1.5. Pearson correlation analysis revealed strong associations between these aroma compounds and major physicochemical parameters (r > 0.80, *p* < 0.01).

Collectively, the six cultivars exhibited distinct morphological and aroma characteristics and generally showed favorable quality traits under the local cultivation conditions. These differences support differentiated market applications: ‘Rainier’ may be suitable for premium fresh consumption, ‘Tieton’ for large-fruit markets, and ‘Lapins’ may have potential for juice processing. The high phenolic content of ‘Hongdeng’ further suggests its potential for the development of functional fruit products. The integration of morphological traits, sugar and organic acid profiles, phenolic composition, volatile compounds, and odor activity values establishes a comprehensive framework for evaluating sweet cherry quality, while the distinct sugar–acid, phenolic, and volatile profiles of the six cultivars offer practical guidance for cultivar selection, quality-oriented breeding in arid regions, orchard planning, standardized fruit grading, regional brand development, and the selection of raw materials for juice and fermented wine production. These findings may facilitate cultivar-specific market positioning and enhance the economic value of sweet cherry production in arid Xinjiang.

## Figures and Tables

**Table 1:** Pearson Correlation Coefficient Matrix Between Basic Physicochemical Indicators and Key Aroma Compounds.

	(E)-2-Hexen-1-ol	β-Ionone	Ethyl 2-Methylpropanoate	Ethyl Octanoate	Benzaldehyde	Isobutanol	Isoamyl Alcohol	Benzyl Alcohol
Fruit longitudinal diameter	0.28	0.15	−0.37	0.11	0.09	0.02	0.1	0.35
Fruit transverse diameter	0.42	0.08	−0.45	0.05	0.03	−0.05	0.02	0.52 *
Stalk length	−0.51 *	−0.22	0.18	−0.17	0.62 **	0.68 **	0.65 **	−0.33
Fruit weight	0.45	0.02	−0.53 *	0.01	−0.04	−0.12	−0.03	0.61 **
Stalk and core weight	0.38	−0.04	−0.58 *	−0.02	−0.1	−0.18	−0.1	0.66 **
Fruit firmness	0.56 *	0.1	−0.61 **	0.08	−0.15	−0.22	−0.14	0.69 **
Soluble solids content	−0.31	−0.19	0.33	−0.05	0.78 **	0.85 **	0.82 **	−0.25
L*	−0.4	−0.25	0.41	−0.09	0.83 **	0.88 **	0.86 **	−0.3
a*	0.12	0.05	−0.22	0.03	0.25	0.18	0.22	0.19
b*	−0.35	−0.21	0.38	−0.07	0.80 **	0.86 **	0.84 **	−0.27

Values are Pearson correlation coefficients (r). * *p* < 0.05, ** *p* < 0.01.

## Data Availability

The original contributions presented in this study are included in the article/supplementary material. Further inquiries can be directed to the corresponding authors.

## Supplementary Materials

The following supporting information can be downloaded at: https://www.mdpi.com/article/10.3390/foods15162809/s1, Table S1: Monthly meteorological indicators of Shache County, Kashgar Prefecture during the sweet cherry growing season (March–June) in 2025; Table S2: Basic Indicators of Different cultivars of Sweet Cherries; Table S3: Organic acids, soluble sugar and individual phenolic compounds in six sweet cherry cultivars; Table S4: Volatile aroma components of different sweet cherry cultivars (μg/L); Table S5: Odour threshold of the studied compounds (mg/L). Figure S1: Monthly meteorological indicators of Shache County, Kashgar Prefecture during the sweet cherry growing season (March–June) in 2025; Figure S2: The chromatograms of the GC-MS analysis.

## Abbreviations

The following abbreviations are used in this manuscript:

HS-SPME	Headspace Solid-Phase Microextraction
GC-MS	Gas Chromatography–Mass Spectrometry
SIM	Selected Ion Monitoring
SCAN	Full-scan
EI	Electron Ionization
NIST	National Institute of Standards and Technology
DVB	Divinylbenzene
PDMS	Polydimethylsiloxane
PTFE	Polytetrafluoroethylene
4M2P	4-methyl-2-pentanol
PVPP	Polyvinylpolypyrrolidone
OAV	Odor Activity Value
ANOVA	Analysis of Variance
SD	Standard Deviation
PCA	Principal Component Analysis
PLS-DA	Partial Least Squares Discriminant Analysis
HSD	Honestly Significant Difference
LOX	Lipoxygenase
HPL	Hydroperoxide Lyase
AAT	Alcohol Acyltransferase
*m*/*z*	mass-to-charge ratio
SPSS	Statistical Product and Service Solutions
N	Newton
μm	micrometer
S	Supplementary
r	Pearson correlation coefficient
