# Supplementary material for: Fruit Quality Assessments of Six Sweet Cherry Cultivars Grown in the Arid Northwest China
Source: Foods. 2026 Aug 12;15(16):2809. doi: 10.3390/foods15162809 (PMC13512657; doi:10.3390/foods15162809)
Supplement: Supplementary file 1 [file foods-15-02809-s001.zip › Table S1.pdf]

Table S1. Monthly meteorological indicators of Shache County, Kashgar Prefecture during the sweet cherry growing season (March–June) in 2025.

Month	Monthly Average Temperature (°C)	Monthly Mean Maximum Temperature (°C)	Monthly Mean Minimum Temperature (°C)	Total Monthly Precipitation (mm)
March	9.8	16	3	0.0
April	17.8	25	10	11.9
May	24.1	31	17	20.6
June	27.2	35	19	23.8

Meteorological data for 2025 were obtained from the historical weather records of Shache County on Tianqi24.com (https://www.tianqi24.com).
