# Supplementary material for: Fruit Quality Assessments of Six Sweet Cherry Cultivars Grown in the Arid Northwest China
Source: Foods. 2026 Aug 12;15(16):2809. doi: 10.3390/foods15162809 (PMC13512657; doi:10.3390/foods15162809)
Supplement: Supplementary file 1 [file foods-15-02809-s001.zip › Table S2.pdf]

Table S2. Basic Indicators of Different cultivars of Sweet Cherries

	Fruit longitudinal diameter (mm)	Fruit transverse diameter (mm)	Stalk length (cm)	Fruit weight (g)	Stalk and core weight (g)	Fruit firmness (N)	Soluble solids content (°Brix)	Total phenolic content (g/L)	*L* *	*a* *	*b* *
HongDeng	21.97 ± 1.26c	27.06 ± 1.29a	2.71 ± 0.65bc	8.89 ± 0.98cd	0.50 ± 0.06bc	10.31 ± 1.41d	16.53 ± 0.12e	0.69 ± 0.01a	24.47 ± 0.76c	19.22 ± 4.32cd	3.96 ± 1.22c
Lapins	24.23 ± 1.24a	24.08 ± 1.28c	3.13 ± 0.51b	8.43 ± 0.95d	0.47 ± 0.05c	12.75 ± 2.91cd	21.03 ± 0.57b	0.50 ± 0.02b	24.82 ± 1.03c	23.96 ± 5.42bc	4.74 ± 1.65c
Summit	23.44 ± 1.21ab	27.00 ± 1.06a	3.04 ± 0.38b	10.59 ± 3.31ab	0.51 ± 0.05bc	10.47 ± 1.67d	17.60 ± 0.53d	0.47 ± 0.01bc	32.81 ± 3.42b	42.23 ± 5.67a	13.78 ± 3.28b
Rainier	22.64 ± 3.02bc	25.01 ± 1.33bc	3.80 ± 0.43a	8.08 ± 0.76d	0.54 ± 0.05b	15.79 ± 2.04b	24.67 ± 0.47a	0.25 ± 0.01d	49.97 ± 7.39a	31.32 ± 9.34b	23.14 ± 3.72a
Van	23.95 ± 1.47a	25.76 ± 1.73b	3.32 ± 0.42b	9.81 ± 1.23bc	0.53 ± 0.09b	16.14 ± 2.67b	18.07 ± 0.51cd	0.44 ± 0.02c	24.81 ± 0.75c	23.80 ± 3.68bc	4.73 ± 1.30c
Tieton	23.97 ± 1.45a	28.11 ± 2.03a	1.95 ± 0.43d	11.37 ± 1.76a	0.67 ± 0.09a	19.57 ± 2.99a	18.77 ± 0.46c	0.47 ± 0.04bc	23.87 ± 1.04c	16.20 ± 5.61d	3.20 ± 1.41c

Different lowercase letters within the same column indicate significant differences among cultivars at $p < 0.05$ based on Tukey's HSD test.
