# Supplementary material for: Fruit Quality Assessments of Six Sweet Cherry Cultivars Grown in the Arid Northwest China
Source: Foods. 2026 Aug 12;15(16):2809. doi: 10.3390/foods15162809 (PMC13512657; doi:10.3390/foods15162809)
Supplement: Supplementary file 1 [file foods-15-02809-s001.zip › Table S3.pdf]

Table S3. Organic acids, soluble sugar and individual phenolic compounds in six sweet cherry cultivars.

	Hongdeng	Lapins	Summit	Rainier	Van	Tieton
Citric acid (g/L)	12.04 ± 0.11b	14.18 ± 0.09a	9.68 ± 0.08c	5.37 ± 0.06d	5.30 ± 0.06d	9.06 ± 0.07c
Tartaric acid (g/L)	0.52 ± 0.03d	0.63 ± 0.03cd	0.91 ± 0.05b	1.43 ± 0.09a	0.77 ± 0.08c	0.97 ± 0.07b
Malic acid (g/L)	15.94 ± 0.10d	14.63 ± 0.11e	16.76 ± 0.09c	20.27 ± 0.13a	11.56 ± 0.08f	17.34 ± 0.11b
Succinic acid (g/L)	2.39 ± 0.01c	2.09 ± 0.01d	3.26 ± 0.02b	4.00 ± 0.03a	2.10 ± 0.02d	2.81 ± 0.02c
Maltose (g/L)	0.07 ± 0.01b	0.06 ± 0.01b	0.06 ± 0.02b	0.08 ± 0.02b	0.06 ± 0.01b	0.70 ± 0.01a
Glucose (g/L)	73.57 ± 5.11b	47.32 ± 3.57d	61.64 ± 4.40c	84.14 ± 3.99a	52.41 ± 4.67cd	65.03 ± 5.03c
Fructose (g/L)	72.62 ± 6.21b	46.97 ± 5.73d	62.43 ± 4.26c	78.39 ± 5.09a	47.58 ± 4.78d	63.38 ± 5.27c
Gallic acid (mg/L)	1.63 ± 0.05bc	1.92 ± 0.05b	1.60 ± 0.04bc	1.60 ± 0.01bc	2.14 ± 0.03ab	2.48 ± 0.07a
Procyanidin B1 (mg/L)	77.46 ± 5.00a	54.82 ± 5.96b	26.81 ± 3.57c	21.78 ± 2.63cd	40.83 ± 3.91bc	12.91 ± 2.07d
Catechin (mg/L)	2.30 ± 0.05d	1.16 ± 0.01e	4.42 ± 0.04b	3.42 ± 0.04c	6.22 ± 0.03a	2.45 ± 0.01d
Chlorogenic acid (mg/L)	44.32 ± 3.77a	32.18 ± 3.54bc	27.25 ± 3.06c	29.86 ± 3.04c	39.98 ± 3.65ab	30.61 ± 2.75bc
Caffeic acid (mg/L)	0.66 ± 0.01b	0.42 ± 0.02c	0.15 ± 0.01d	0.16 ± 0.01d	0.91 ± 0.02a	0.06 ± 0.01d
Epicatechin (mg/L)	3.07 ± 0.15bc	2.12 ± 0.12cd	2.63 ± 0.17bc	3.17 ± 0.10b	5.23 ± 0.19a	0.91 ± 0.08d
p-Coumaric acid (mg/L)	68.72 ± 3.33a	66.68 ± 3.79a	32.01 ± 3.04cd	30.03 ± 2.99d	37.34 ± 3.52c	44.88 ± 3.75b
Rutin (mg/L)	28.07 ± 2.90bc	34.49 ± 2.85ab	35.18 ± 2.77a	30.45 ± 2.91bc	25.85 ± 3.01c	27.42 ± 2.48bc
Quercetin (mg/L)	2.20 ± 0.05d	1.55 ± 0.06e	2.60 ± 0.05c	2.94 ± 0.02b	2.35 ± 0.03cd	4.10 ± 0.01a
Kaempferol (mg/L)	2.82 ± 0.01a	2.72 ± 0.02a	1.31 ± 0.01c	1.46 ± 0.02b	2.46 ± 0.03a	1.19 ± 0.01d

Different lowercase letters within the same row indicate significant differences among cultivars at $p < 0.05$ based on Tukey's HSD test.
