# Supplementary material for: Fruit Quality Assessments of Six Sweet Cherry Cultivars Grown in the Arid Northwest China
Source: Foods. 2026 Aug 12;15(16):2809. doi: 10.3390/foods15162809 (PMC13512657; doi:10.3390/foods15162809)
Supplement: Supplementary file 1 [file foods-15-02809-s001.zip › Table S4.pdf]

Table S4. Volatile aroma components of different sweet cherry cultivars (μg/L)

Compound Category	Aroma compounds	Hongdeng	Lapins	Summit	Rainier	Van	Tieton
Esters	Ethyl isobutyrate	0.52 ± 0.08 ab	0.31 ± 0.04 c	0.29 ± 0.01 c	0.38 ± 0.06 bc	0.35 ± 0.10 bc	0.73 ± 0.61 a
	Ethyl 2-methylbutanoate	36.07 ± 10.78 e	46.70 ± 2.67 d	48.40 ± 1.89 d	308.51 ± 26.75 a	99.41 ± 30.42 b	87.88 ± 27.18 c
	Ethyl 2-methylpropanoate	11.36 ± 1.95 a	7.51 ± 0.91 b	4.99 ± 0.02 cd	6.98 ± 2.03 bc	4.72 ± 0.37 d	4.46 ± 0.06 d
	Ethyl acetate	284.17 ± 40.70 b	504.51 ± 488.79 a	191.89 ± 2.79 d	263.59 ± 12.89 bc	213.07 ± 18.38 cd	214.54 ± 10.81 cd
	Isoamyl acetate	57.56 ± 0.41 ab	58.09 ± 0.18 a	58.02 ± 0.18 a	55.51 ± 0.42 c	57.02 ± 0.71 bc	57.39 ± 0.22 abc
	Ethyl hexanoate	1.61 ± 0.37 a	0.81 ± 0.09 c	0.88 ± 0.02 bc	1.23 ± 0.06 ab	0.94 ± 0.17 bc	1.18 ± 0.45 abc
	Methyl octanoate	3.71 ± 0.01 ab	3.70 ± 0.01 b	3.70 ± 0.01 b	3.71 ± 0.01 ab	3.71 ± 0.01 ab	3.72 ± 0.01 a
	Isoamyl hexanoate	3.54 ± 0.02 bc	3.52 ± 0.00 c	3.55 ± 0.01 bc	3.77 ± 0.02 a	3.68 ± 0.05 ab	3.76 ± 0.07 a
	Ethyl nonanoate	4.18 ± 0.01 ab	4.16 ± 0.01 b	4.17 ± 0.01 ab	4.25 ± 0.01 a	4.17 ± 0.01 ab	4.18 ± 0.01 ab
	Ethyl hexanoate	29.94 ± 0.06 a	29.85 ± 0.01 bc	29.78 ± 0.01 c	29.84 ± 0.01 bc	29.88 ± 0.07 abc	29.88 ± 0.04 abc
	Ethyl decanoate	144.15 ± 0.10 c	144.01 ± 0.05 d	144.29 ± 0.04 b	144.51 ± 0.17 a	144.17 ± 0.12 c	144.64 ± 0.15 a
	Ethyl butanoate	310.75 ± 0.60 a	309.77 ± 0.05 bc	309.30 ± 0.02 d	309.67 ± 0.01 c	309.39 ± 0.08 cd	309.34 ± 0.06 cd
	Geranyl acetate	5.56 ± 0.00 b	5.56 ± 0.01 b	5.56 ± 0.01 b	5.58 ± 0.01 a	5.56 ± 0.01 b	5.56 ± 0.01 b
	Methyl salicylate	4.59 ± 0.01 bc	4.58 ± 0.01 c	4.59 ± 0.04 bc	4.67 ± 0.06 a	4.63 ± 0.01 ab	4.63 ± 0.01 ab
	Ethyl salicylate	3.23 ± 0.01 c	3.24 ± 0.01 bc	3.24 ± 0.01 bc	3.25 ± 0.01 a	3.26 ± 0.01 a	3.26 ± 0.01 a
	Phenethyl acetate	17.32 ± 0.03 b	17.26 ± 0.01 c	17.26 ± 0.01 c	17.40 ± 0.01 a	17.38 ± 0.05 a	17.40 ± 0.03 a
	Ethyl dodecanoate	27.09 ± 0.00 ab	27.09 ± 0.01 b	27.09 ± 0.01 b	27.09 ± 0.01 ab	27.09 ± 0.01 ab	27.10 ± 0.01 a
	Ethyl octanoate	109.51 ± 0.03 a	109.48 ± 0.01 b	109.48 ± 0.01 b	109.48 ± 0.01 b	109.48 ± 0.02 b	109.53 ± 0.03 a
	Subtotal	1054.86 ± 55.17 c	1280.14 ± 492.83 ab	966.47 ± 5.04 d	1299.42 ± 42.51 a	1037.91 ± 50.58 c	1029.19 ± 39.72 c
	Kinds	18	18	18	18	18	18
	Proportion	5.36%	7.41%	5.08%	1.93%	3.90%	3.43%
Alcohols	Isobutanol	3383.12 ± 982.52 d	4112.61 ± 230.03 d	4091.45 ± 132.13 d	25226.31 ± 1897.79 a	8230.66 ± 2408.60 b	7246.23 ± 2222.29 c
	Isoamyl alcohol	1473.08 ± 563.02 e	2199.82 ± 118.77 d	3096.70 ± 134.85 c	19447.61 ± 1349.23 a	5034.80 ± 1820.65 b	4122.88 ± 1135.28 c
	3-methyl-1-pentanol	75.82 ± 45.80 b	35.72 ± 6.89 c	1.61 ± 0.01 d	152.65 ± 16.12 a	51.82 ± 0.01 c	178.44 ± 45.92 a
	(E)-3-hexen-1-ol	195.11 ± 80.83 a	3.07 ± 0.23 c	1.50 ± 0.14 c	7.17 ± 1.41 c	3.17 ± 1.31 c	8.50 ± 2.68 c
	(Z)-3-hexen-1-ol	54.03 ± 24.58 b	8.05 ± 3.72 c	7.54 ± 0.80 c	25.38 ± 3.66 bc	11.25 ± 4.87 c	197.98 ± 283.01 a
	1-butanol	2750.30 ± 18.42 a	2787.09 ± 10.73 a	2798.82 ± 0.55 a	2739.88 ± 3.25 a	2741.71 ± 26.90 a	2744.22 ± 17.06 a
	(Z)-3-hexen-1-ol	7.53 ± 4.04 ab	6.41 ± 3.78 ab	5.29 ± 0.40 b	11.43 ± 2.68 a	5.41 ± 0.51 b	8.50 ± 1.85 ab
	1-octen-3-ol	4.54 ± 0.01 b	4.54 ± 0.01 b	4.54 ± 0.01 b	4.58 ± 0.01 a	5.00 ± 0.12 a	5.18 ± 0.13 a
	1-heptanol	10.56 ± 0.14 b	10.40 ± 0.03 b	10.35 ± 0.02 b	11.41 ± 0.09 a	10.71 ± 0.23 ab	11.16 ± 0.33 a
	2-ethyl-1-hexanol	4.50 ± 0.13 ab	4.45 ± 0.21 ab	4.31 ± 0.03 b	6.66 ± 3.42 a	4.40 ± 0.09 b	4.73 ± 0.25 ab
	Linalool	4.55 ± 0.01 d	4.98 ± 0.39 cd	6.18 ± 0.08 bc	19.01 ± 0.91 a	6.93 ± 0.74 b	7.38 ± 2.30 b

	1-octanol	10.57 ± 0.10 b	10.73 ± 0.25 ab	10.85 ± 0.04 ab	11.74 ± 0.12 a	10.92 ± 0.30 ab	10.68 ± 0.19 ab
	1-decanol	78.65 ± 0.01 a	78.65 ± 0.01 a	78.64 ± 0.01 a	78.68 ± 0.24 a	78.64 ± 0.14 a	78.64 ± 0.01 a
	Nerol	3.14 ± 0.05 c	3.07 ± 0.03 c	3.13 ± 0.01 c	3.73 ± 0.08 b	4.23 ± 0.24 a	4.18 ± 0.17 a
	Geraniol	8.06 ± 0.11 c	7.91 ± 0.07 c	8.04 ± 0.02 c	9.26 ± 0.16 b	10.30 ± 0.50 a	10.20 ± 0.35 a
	Benzyl alcohol	1840.52 ± 668.01 b	198.84 ± 2.85 d	882.81 ± 51.45 c	2425.26 ± 29.02 b	1986.76 ± 675.94 b	4879.21 ± 983.99 a
	Phenethyl alcohol	1967.31 ± 9.38 c	1951.94 ± 1.19 c	1958.93 ± 1.42 c	2057.72 ± 1.40 a	1972.26 ± 18.66 bc	1987.19 ± 9.63 b
	(E)-2-hexen-1-ol	760.27 ± 261.69 b	294.97 ± 10.38 d	80.44 ± 5.19 e	703.95 ± 36.60 b	404.78 ± 129.23 c	1379.88 ± 265.61 a
	1-propanol	2108.04 ± 150.32 a	2037.10 ± 25.04 ab	1996.88 ± 111.16 bc	1896.52 ± 6.92 d	1962.90 ± 60.08 cd	1947.97 ± 118.73 cd
	Citronellol	6.90 ± 0.02 c	6.89 ± 0.01 c	6.92 ± 0.01 bc	7.11 ± 0.02 a	6.93 ± 0.01 bc	6.95 ± 0.01 b
	Subtotal	14746.60 ± 2809.19 c	13767.25 ± 414.60 c	15054.93 ± 438.30 c	54846.05 ± 3353.13 a	22543.57 ± 5148.99 b	24840.09 ± 5089.79 b
	Kinds	20	20	20	20	20	20
	Proportion	74.94%	79.64%	79.12%	81.37%	84.63%	82.85%
Aldehyde	Nonanal	27.17 ± 8.86 b	11.53 ± 0.26 d	4.24 ± 0.16 e	25.31 ± 1.22 b	15.19 ± 4.37 c	48.12 ± 8.96 a
	Decanal	0.59 ± 0.15 ab	0.52 ± 0.10 b	0.49 ± 0.08 b	0.79 ± 0.17 a	0.62 ± 0.06 ab	0.75 ± 0.29 a
	Benzaldehyde	2871.09 ± 896.20 c	1369.42 ± 81.64 d	2015.16 ± 212.34 c	9913.01 ± 690.62 a	2074.69 ± 712.11 c	3036.54 ± 806.60 b
	Octanal	10.24 ± 3.93 a	2.57 ± 0.73 c	2.34 ± 0.10 c	5.07 ± 0.35 b	4.14 ± 1.17 bc	7.69 ± 2.30 ab
	Subtotal	2909.08 ± 909.15 b	1384.05 ± 82.73 c	2022.23 ± 212.68 c	9944.18 ± 692.36 a	2094.64 ± 717.72 c	3093.10 ± 818.14 b
	Kinds	4	4	4	4	4	4
	Proportion	14.78%	8.01%	10.63%	14.75%	7.86%	10.32%
Acids	Hexanoic acid	200.85 ± 10.54 c	188.06 ± 0.30 c	227.36 ± 3.05 b	263.15 ± 23.83 a	209.46 ± 14.73 c	266.76 ± 16.38 a
	Octanoic acid	194.64 ± 0.40 a	194.29 ± 0.42 a	194.02 ± 0.03 a	195.23 ± 1.23 a	196.72 ± 3.50 a	194.89 ± 0.59 a
	Decanoic acid	62.88 ± 0.16 a	62.78 ± 0.10 ab	62.73 ± 0.03 b	63.01 ± 0.31 a	62.79 ± 0.05 ab	63.00 ± 0.18 a
	Isobutyric acid	107.38 ± 2.68 a	105.72 ± 0.33 b	106.61 ± 1.58 ab	106.26 ± 0.15 ab	105.77 ± 0.77 b	105.85 ± 0.73 b
	Benzoic acid	155.27 ± 69.81 b	64.21 ± 5.19 c	149.76 ± 27.14 b	361.86 ± 14.83 a	126.99 ± 79.30 b	119.25 ± 26.12 b
	Isovaleric acid	136.67 ± 0.43 bc	136.16 ± 0.01 c	137.74 ± 0.12 b	139.19 ± 0.96 a	137.02 ± 0.59 b	139.33 ± 0.66 a
	Subtotal	857.69 ± 84.02 b	751.21 ± 6.35 c	878.22 ± 31.96 b	1128.70 ± 41.30 a	838.75 ± 98.94 b	889.08 ± 44.65 b
	Kinds	7	7	7	7	7	7
	Proportion	4.36%	4.35%	4.62%	1.67%	3.15%	2.97%
Phenols	Phenol	22.72 ± 1.18 bc	20.29 ± 0.48 c	22.50 ± 0.31 bc	44.15 ± 1.21 a	26.65 ± 4.01 b	28.55 ± 2.55 b
	Subtotal	22.72 ± 1.18 bc	20.29 ± 0.48 c	22.50 ± 0.31 bc	44.15 ± 1.21 a	26.65 ± 4.01 b	28.55 ± 2.55 b
	Kinds	1	1	1	1	1	1
	Proportion	0.12%	0.12%	0.12%	0.07%	0.10%	0.10%
Terpenes	(R)-(-)-4-terpineol	2.82 ± 0.30 ab	2.66 ± 0.05 b	2.71 ± 0.05 b	2.73 ± 0.11 b	2.91 ± 0.32 ab	3.32 ± 0.53 a
	α-Terpineol	3.19 ± 0.06 d	3.22 ± 0.03 d	3.50 ± 0.02 c	6.74 ± 0.37 a	3.80 ± 0.20 b	4.03 ± 0.11 b
	D-Limonene	9.32 ± 1.55 d	11.26 ± 0.36 cd	13.68 ± 0.40 c	58.30 ± 3.64 a	19.02 ± 5.02 b	16.46 ± 3.09 b
	Subtotal	15.33 ± 1.91 d	17.15 ± 0.44 cd	19.88 ± 0.48 c	67.78 ± 4.12 a	25.73 ± 5.54 b	23.81 ± 3.73 b
	Kinds	3	3	3	3	3	3
	Proportion	0.08%	0.10%	0.10%	0.10%	0.10%	0.08%

Other substance	Furfural	44.38 ± 4.92 bc	41.01 ± 2.96 c	38.91 ± 0.90 c	48.51 ± 4.08 ab	42.48 ± 3.28 c	51.14 ± 7.30 a
	2-methoxy-3-(2-methylpropyl)pyrazine	2.40 ± 0.01 b	2.40 ± 0.01 b	2.40 ± 0.01 b	2.41 ± 0.01 a	2.40 ± 0.01 ab	2.40 ± 0.01 ab
	β-Ionone	3.38 ± 0.01 ab	3.38 ± 0.01 b	3.38 ± 0.01 b	3.38 ± 0.01 b	3.39 ± 0.01 ab	3.40 ± 0.01 a
	cis-4-methyl-5-butyldihydrofuran-2(3H)-one	20.12 ± 0.24 cd	19.99 ± 0.11 d	20.18 ± 0.01 cd	22.61 ± 0.37 a	21.49 ± 0.64 b	20.58 ± 0.16 c
	Subtotal	70.28 ± 5.17 bc	66.78 ± 3.07 c	64.87 ± 0.91 c	76.91 ± 4.46 a	69.76 ± 3.93 bc	77.51 ± 7.47 a
	Kinds	4	4	4	4	4	4
	Proportion	0.36%	0.39%	0.34%	0.11%	0.26%	0.26%
	Total	19676.57 ± 3865.78 c	17286.86 ± 1000.51 c	19029.09 ± 689.66 c	67407.18 ± 4139.09 a	26637.01 ± 6029.70 b	29981.33 ± 6006.05 b
	Total Kinds	56	56	56	56	56	56

Different lowercase letters within the same row indicate significant differences among cultivars at $p < 0.05$ based on Tukey's HSD test.
