# Supplementary material for: Fruit Quality Assessments of Six Sweet Cherry Cultivars Grown in the Arid Northwest China
Source: Foods. 2026 Aug 12;15(16):2809. doi: 10.3390/foods15162809 (PMC13512657; doi:10.3390/foods15162809)
Supplement: Supplementary file 1 [file foods-15-02809-s001.zip › Table S5.pdf]

Table S5. Odour threshold of the studied compounds (mg/L)

Compound Category	Aroma compounds	CAS#	RI[a]	RI-NIST[b]	Odour threshold (mg/L)	Hongdeng	Lapins	Summit	Rainier	Van	Tieton
Esters	Ethyl 2-methylbutanoate	7452-79-1	820	820	0.05	0.72 ± 0.22 e	0.93 ± 0.05 d	0.97 ± 0.04 d	6.17 ± 0.53 a	1.99 ± 0.61 b	1.76 ± 0.54 c
	Ethyl 2-methylpropanoate	97-62-1	956	955	0.001	11.36 ± 1.95 a	7.51 ± 0.91 b	4.99 ± 0.02 cd	6.98 ± 2.03 bc	4.72 ± 0.37 d	4.46 ± 0.06 d
	Methyl octanoate	111-11-5	985	986	0.001	3.71 ± 0.01 ab	3.70 ± 0.01 b	3.70 ± 0.01 b	3.71 ± 0.01 ab	3.71 ± 0.01 ab	3.72 ± 0.01 a
	Isoamyl hexanoate	2198-61-0	1468	1468	0.001	3.54 ± 0.02 c	3.52 ± 0.01 c	3.55 ± 0.01 bc	3.77 ± 0.02 a	3.68 ± 0.05 b	3.76 ± 0.07 a
	Ethyl nonanoate	123-29-5	1788	1751	0.001	4.18 ± 0.01 ab	4.16 ± 0.01 b	4.17 ± 0.01 ab	4.25 ± 0.01 a	4.17 ± 0.01 ab	4.18 ± 0.01 ab
	Ethyl hexanoate	123-66-0	1221	1220	0.014	2.14 ± 0.01 a	2.13 ± 0.01 b	2.13 ± 0.00 b	2.13 ± 0.01 b	2.13 ± 0.01 b	2.13 ± 0.01 b
	Ethyl butanoate	105-54-4	1027	1028	0.02	15.54 ± 0.03 a	15.49 ± 0.01 b	15.46 ± 0.01 d	15.48 ± 0.01 bc	15.47 ± 0.01 cd	15.47 ± 0.01 cd
	Geranyl acetate	105-87-3	1765	1765	0.001	5.56 ± 0.01 b	5.56 ± 0.01 b	5.56 ± 0.01 b	5.58 ± 0.01 a	5.56 ± 0.01 b	5.56 ± 0.01 b
	Ethyl dodecanoate	106-33-2	1843	1842	0.001	27.09 ± 0.01 b	27.09± 0.01 b	27.09 ± 0.01 b	27.09 ± 0.01 b	27.09 ± 0.01 b	27.10 ± 0.01 a
	Ethyl octanoate	106-32-1	1435	1436	0.005	21.90 ± 0.01 a	21.90 ± 0.01 a	21.90 ± 0.01 a	21.89 ± 0.01 b	21.90 ± 0.01 a	21.91 ± 0.01 a
Alcohols	Isobutanol	78-83-1	1100	1099	0.5	6.77 ± 1.97 d	8.23 ± 0.46 d	8.18 ± 0.26 d	50.45 ± 3.80 a	16.46 ± 4.82 b	14.49 ± 4.44 c
	Isoamyl alcohol	123-51-3	1206	1205	0.5	2.95 ± 1.13 e	4.40 ± 0.24 d	6.19 ± 0.27 c	38.90 ± 2.70 a	10.07 ± 3.64 b	8.25 ± 2.27 c
	1-butanol	71-36-3	1158	1163	2	1.38 ± 0.01 a	1.39 ± 0.01 a	1.40 ± 0.01 a	1.37 ± 0.01 b	1.37 ± 0.01 b	1.37 ± 0.01 b
	(Z)-3-hexen-1-ol	928-94-9	865	866	0.0008	9.41 ± 5.05 ab	8.01 ± 4.73 ab	6.61 ± 0.50 b	14.28 ± 3.35 a	6.77 ± 0.64 b	10.62 ± 2.31 ab
	Nerol	106-25-2	1771	1770	0.001	3.14 ± 0.05 c	3.07 ± 0.03 c	3.13 ± 0.01 c	3.73 ± 0.08 b	4.23 ± 0.24 a	4.18 ± 0.17 a

	(E)-2-hexen-1-ol	928-95-0	875	876	0.0004	1900.68 ± 654.23 b	737.42 ± 25.96 d	201.10 ± 12.96 e	1759.86 ± 91.50 b	1011.95 ± 23.09 c	3449.70 ± 664.03 a
Aldehyde	Nonanal	124-19-6	1403	1441	0.01	2.72 ± 0.89 b	1.15 ± 0.03 d	0.42 ± 0.02 e	2.53 ± 0.12 b	1.52 ± 0.44 c	4.81 ± 0.90 a
	Furfural	98-01-1	830	831	0.03	1.48 ± 0.16 ab	1.37 ± 0.10 b	1.30 ± 0.03 b	1.62 ± 0.14 a	1.42 ± 0.11 ab	1.70 ± 0.24 a
	Benzaldehyde	100-52-7	1530	1538	1	2.87 ± 0.90 c	1.37 ± 0.08 d	2.02 ± 0.21 c	9.91 ± 0.69 a	2.07 ± 0.71 c	3.04 ± 0.81 b
	Octanal	124-13-0	980	981	0.0007	14.63 ± 5.62 a	3.68 ± 1.05 c	3.34 ± 0.14 c	7.25 ± 0.50 b	5.91 ± 1.67 bc	10.98 ± 3.28 a
Acids	Isobutyric acid	79-31-2	790	791	0.08	1.34 ± 0.03 a	1.32 ± 0.01 b	1.33 ± 0.02 ab	1.33 ± 0.01 ab	1.32 ± 0.01 b	1.32 ± 0.01 b
Other substance	2-methoxy-3-(2-methylpropyl)pyrazine	24683-00-9	1511	1510	0.001	2.40 ± 0.01 b	2.40 ± 0.01 b	2.40 ± 0.01 b	2.41 ± 0.01 a	2.40 ± 0.01 b	2.40 ± 0.01 b
	β-Ionone	79-77-6	1425	1427	0.000003	1127.36 ± 1.66 b	1125.92 ± 0.79 c	1125.38 ± 0.21 c	1125.67 ± 1.64 c	1128.61 ± 1.54 ab	1132.72 ± 1.75 a
	cis-4-methyl-5-butyldihydrofuran-2(3H)-one	55013-32-6	1885	1886	0.001	20.12 ± 0.24 cd	19.99 ± 0.11 d	20.18 ± 0.01 cd	22.61 ± 0.37 a	21.49 ± 0.64 b	20.57 ± 0.16 c

Annotation: aLRI, retention index values were calculated using n-alkanes. bLRI-NIST, retention index values from NIST database. Compounds were positively identified if experimental LRI matched reference values (LRI-NIST) within ±10-20 units, different letters in the same row indicate significant differences among treatments ($p < 0.05$). Odour-threshold values were retrieved from Ferreira et al., 2000, Ribéreau-Gayon et al., 2006 and the NIST Chemistry WebBook.
